# Fatty liver is associated with significant liver inflammation and increases the burden of advanced fibrosis in chronic HBV infection

**DOI:** 10.1186/s12879-023-08632-y

**Published:** 2023-09-28

**Authors:** Yi-Ning Dai, Cheng-Fu Xu, Hong-Ying Pan, Mei-Juan Chen, Chao-Hui Yu

**Affiliations:** 1 Department of Gastroenterology, The First Affiliated Hospital, Zhejiang University School of Medicine, Hangzhou, 310003 Zhejiang Province China; 2 Center for General Practice Medicine, Department of Infectious Diseases, Zhejiang Provincial People’s Hospital (Affiliated People’s Hospital, Hangzhou Medical College), Hangzhou, 310014 Zhejiang Province China

**Keywords:** Chronic HBV infection, Non-alcoholic fatty liver disease, Significant liver inflammation, Significant fibrosis, Advanced liver fibrosis, Natural history, Predictive model

## Abstract

**Background:**

Concurrent non-alcoholic fatty liver disease (NAFLD) is common in patients with chronic HBV infection. But the impact of fatty liver on the histologic progression of HBV infection remains controversial.

**Methods:**

Consecutive HBV-infected patients who underwent liver biopsy between 2016 and 2021 were included. Alcohol consumption and other types of viral hepatitis were excluded. All biopsies were scored for grading and staging by Scheuer’s score, and the steatosis was scored as an estimate of the percentage of liver parenchyma replaced by fat. Logistic regression analyses were applied to assess the associated factors for significant liver inflammation (G ≥ 2), significant fibrosis (S ≥ 2) and advanced fibrosis (S ≥ 3).

**Results:**

Among the 871 HBV-infected patients, hepatic steatosis was prevalent in 255 patients (29.28%). Significant liver inflammation was present in 461 patients (52.93%). Significant fibrosis was observed in 527 patients (60.51%), while advanced liver fibrosis was observed in 171 patients (19.63%). Patients with concomitant NAFLD were more likely to have significant liver inflammation and advanced fibrosis. Fatty liver was an independent risk factor for significant liver inflammation (OR: 2.117, 95% CI: 1.500-2.988), but it could not predict the development of fibrosis. Especially, in HBV-infected patients with persistent normal ALT (immune tolerant and inactive carrier phase), the presence of significant liver inflammation was higher in NAFLD than those without NAFLD. The prevalence of advanced liver fibrosis was higher in NAFLD than non-NAFLD only in the immune tolerant phase, while NAFLD did not increase fibrosis burden in other stages of HBV infection. We developed a predictive model for significant liver inflammation with the area under receiver operating characteristic curve (AUROC) of 0.825, and a model for significant fibrosis with the AUROC of 0.760.

**Conclusions:**

NAFLD is independently associated with significant liver inflammation, and increases the burden of advanced liver fibrosis in HBV-infected patients. The influence of NAFLD on the degree of liver inflammation and fibrosis is different in distinct clinical phases of chronic HBV infection.

## Background

With the dramatic rise in the prevalence of non-alcoholic fatty liver disease (NAFLD), it frequently coexists with other conditions such as alcohol consumption and viral hepatitis. In the meantime, chronic hepatitis B (CHB) caused by hepatitis B virus (HBV) infection is still one of the most common causes of chronic liver diseases in China [1]. Consequently, CHB and NAFLD are frequently observed together with an estimated 30% having hepatic steatosis among those with CHB [2].

Until now, the interplay between the two diseases has not been thoroughly evaluated. Several studies revealed that hepatic steatosis in chronic HBV infection did not appear to affect the severity of liver histology [3, 4]. Conversely, some recent researches concluded steatosis was associated with advanced fibrosis in CHB [5–8]. Therefore, the effect of fatty liver on the natural history of chronic HBV infection still remains controversial [9].

With this background, we aim to compare the histologic differences between simple chronic HBV infection and HBV infection with concomitant NAFLD, and to study whether fatty liver predict severe liver histology like significant liver inflammation or fibrosis in chronic HBV infection. Furthermore, noninvasive models were developed to accurately identify significant liver inflammation and significant fibrosis in this study.

## Methods

### Study design and patients

A retrospective cross-sectional study was conducted at Zhejiang Provincial People’s Hospital. We included patients with chronic HBV infection over 18 years-old who underwent a liver biopsy between 2016 and 2021. The exclusion criteria were as follows: (1) excessive alcohol consumption (ethanol consumption more than 140 g in men and 70 g in women per week); (2) other types of viral hepatitis (e.g., chronic hepatitis C virus infection); (3) other causes of liver injury (e.g., drug-induced liver disease, autoimmune liver disease, or hereditary disorders); (4) pregnancy, malignancy, severe cardiopulmonary disorders, or renal dysfunction.

The natural history of chronic HBV infection has been divided into four clinical phases as follows, taking into account the clinical data of patients including presence of hepatitis B e antigen (HBeAg), Hepatitis B virus DNA (HBV DNA) levels and alanine aminotransferase (ALT) values [10]. (1) HBeAg-positive chronic HBV infection, i.e. “immune tolerant” phase: positive serum HBeAg, very high levels of HBV DNA and ALT persistently within the normal range (upper limit of normal (ULN) of 40 IU/ml). (2) HBeAg-positive CHB: positive serum HBeAg, high levels of HBV DNA and abnormal ALT values. (3) HBeAg-negative chronic HBV infection, i.e. “inactive carrier” phase: negative serum HBeAg, undetectable or low (< 2000 IU/ml) HBV DNA levels and normal ALT; Some patients in this phase may have serum HBV DNA levels > 2000 IU/ml (usually < 20,000 IU/ml) accompanied by persistently normal ALT. (4) HBeAg-negative CHB: negative serum HBeAg, moderate to high levels of HBV DNA, and elevated or fluctuating ALT levels.

This study was approved by the Ethics Committee of People’s Hospital of Hangzhou Medical College and followed the guidelines for studies in humans. Informed consents were obtained from all subjects.

### Clinical data and liver biopsy

Clinical, demographic and laboratory data were collected from the medical records of patients. Complete blood counts, biochemical and virological (HBV DNA, the positivity of HBeAg) data were recorded as the closest results to the date on which liver biopsy was performed.

Skilled doctors performed percutaneous liver biopsy using the MAX-CORE Disposable Core Biopsy Instrument (Bard Peripheral Vascular, Inc., Mexico). The specimens were fixed, paraffin-embedded, and stained by haematoxylin and eosin (H&E) and Masson’s trichrome for further pathological evaluation by an experienced liver pathologist. All liver biopsy slides should be qualified for scoring of histologic features.

A threshold of 5% macrovesicular steatosis made a diagnosis of NAFLD [11]. Steatosis was graded as the percentage of liver parenchyma replaced by fat: (1) 5 − 33%, (2) 34 − 66%, or (3) more than 66% [12]. Lobular inflammation was scored on a scale of 0–3: (0) none, (1) mild, (2) moderate, and (3) many. The degree of portal inflammation and hepatocellular ballooning were divided as: (0) none, (1) mild inflammation or few balloon cells, and (2) prominent inflammation or ballooning. Liver inflammation (G0 to G4) and fibrosis (S0 to S4) were assessed according to the Scheuer scoring system [13]. The grades of liver inflammation were classified into the following 5 stages: G0, no inflammation; G1, inflammatory but no necrosis; G2, focal necrosis or acidophil bodies; G3, severe focal cell damage; and G4, widely bridging necrosis and piecemeal necrosis. Significant liver inflammation was defined as inflammation grade of G2 to G4. Liver fibrosis was scored as follows: S0, no fibrosis; S1, portal fibrosis without septa; S2, portal fibrosis with rare septa; S3, numerous septa without cirrhosis; and S4, cirrhosis. Fibrotic stage of S2 to S4 was considered as significant fibrosis, while fibrotic stage of S3 to S4 was defined as advanced fibrosis [14].

### Statistical analysis

Statistical analyses were performed by SPSS (version 23) and Python 3.7. Continuous variables are described as the mean ± standard deviation (SD), and categorical variables are presented as numbers (percentages). Propensity score-matching (PSM) was used to adjust the potential confounding factors including age, gender, HBV DNA and the positivity of HBeAg. When evaluating differences between groups, the t-test or the chi-squared test was applied. A two-sided P value < 0.05 was considered statistically significant.

Logistic analysis was applied to assess the risk factors for significant liver inflammation, significant fibrosis and advanced fibrosis. The associated factors observed in the univariate analysis were utilized for model training. The establishments of models were based on the Scikit-Learn package of Scientific Python 3.7 libraries. A binary logistic regression was performed to predict the probability of significant liver inflammation and significant fibrosis. We used the LIBLINEAR library to carry out the computation. Area under receiver operating characteristic curve (AUROC) was used to evaluate the predictive accuracy of the model.

## Results

### Study Population

A total of 871 patients with chronic HBV infection were included in this study. The mean age of the study population was 39.14 (± 9.93) years, and 586 (67.28%) were male patients. Among them, 352 patients (40.41%) were HBeAg-positive. Majority of the patients (93.92%) did not receive any antiviral therapy. Hepatic steatosis was present in 255 patients (29.28%): 219 grade 1 steatosis, 33 grade 2 steatosis, and 3 grade 3 steatosis. Significant hepatic inflammation was present in 461 patients (52.62%). Significant fibrosis was observed in 527 patients (60.51%), while advanced liver fibrosis was observed in 171 patients (19.63%).

### Comparisons between HBV-infected patients with and without hepatic steatosis

The characteristics of HBV-infected patients with and without hepatic steatosis are exhibited in Table 1. Compared with patients without fatty liver, chronic HBV infection with concurrent NAFLD were more likely to be male, HBeAg-negative, and to have hypertension and type 2 diabetes mellitus (T2DM). Furthermore, they had significantly higher height, weight, body mass index (BMI) and higher levels of white blood cells (WBC), albumin, total bilirubin, ALT, GGT and ALP.

**Table 1:** Clinical characteristics of HBV-infected patients with and without hepatic steatosis

	HBV infection (N = 616)	HBV infection + NAFLD (N = 255)	P value
Age	38.87 ± 10.19	39.78 ± 9.25	0.222
Gender (M/F)	366/250	220/35	**< 0.001**
Height	164.63 ± 8.23	167.63 ± 7.97	**< 0.001**
Weight	60.69 ± 10.14	73.00 ± 10.71	**< 0.001**
BMI	22.33 ± 2.98	25.98 ± 3.55	**< 0.001**
WBC	5.61 ± 1.51	6.00 ± 1.37	**< 0.001**
PLT	184.83 ± 52.47	191.60 ± 52.89	0.084
ALB	43.77 ± 3.57	44.54 ± 3.40	**0.003**
GLB	29.22 ± 3.93	29.02 ± 4.00	0.482
Tbil	15.23 ± 6.22	16.51 ± 6.29	**0.006**
ALT	38.62 ± 32.75	52.02 ± 37.48	**< 0.001**
AST	33.30 ± 19.40	35.81 ± 17.58	0.075
GGT	27.24 ± 26.64	42.89 ± 36.26	**< 0.001**
ALP	81.10 ± 24.27	89.68 ± 25.51	**< 0.001**
HBV DNA (Log_10_ IU/mL)	4.55 ± 2.66	4.23 ± 2.52	0.093
HbeAg (+)	264 (42.86%)	88 (34.51%)	**0.022**
Anti-viral therapy	36 (5.84%)	17 (6.67%)	0.644
Hypertension	29 (4.71%)	32 (12.55%)	**< 0.001**
T2DM	7 (1.14%)	13 (5.10%)	**< 0.001**

NAFLD: Non-alcoholic fatty liver disease; M: Male; F: Female; BMI: Body mass index; WBC: White blood cell count; PLT: Platelet; ALB: Albumin; GLB: Globulin; Tbil: Total bilirubin; ALT: Alanine aminotransferase; AST: Aspartate transferase; GGT: Gamma-glutamyltransferase; ALP: Alkaline phosphatase; HBV: Hepatitis B virus; HbeAg: hepatitis B e antigen; T2DM: Type 2 diabetes mellitus

Compared to HBV-infected patients without fatty liver, chronic HBV infection with concurrent NAFLD had a higher severity of hepatic steatosis, hepatic inflammation especially portal inflammation, and hepatic ballooning (Table 2). There were no significant differences based on the grades of lobular inflammation or fibrosis. The prevalence of significant fibrosis was similar between the two groups. However, there was borderline difference in the presence of advanced liver fibrosis (*P* = 0.049).

**Table 2:** Histologic characteristics of HBV-infected patients with and without hepatic steatosis

	HBV infection (N = 616)	HBV infection + NAFLD (N = 255)	P value
Steatosis			**< 0.001**
0	616 (100%)	0	
1	0	219 (85.88%)	
2	0	33 (12.94%)	
3	0	3 (1.18%)	
Lobular inflammation			0.323
0	1 (0.16%)	0	
1	389 (63.15%)	150 (58.82%)	
2	210 (34.09%)	93 (36.47%)	
3	15 (2.44%)	12 (4.71%)	
4	1 (0.16%)	0	
Portal inflammation			**< 0.001**
1	360 (58.44%)	101 (39.61%)	
2	227 (36.85%)	127 (49.80%)	
3	26 (4.22%)	26 (10.20%)	
4	3 (0.49%)	1 (0.39%)	
Hepatic ballooning			
0	608 (98.70%)	239 (93.73%)	
1	8 (1.30%)	9 (3.53%)	**< 0.001**
2	0	7 (2.75%)	
3	0	0	
G			**< 0.001**
1	318 (51.62%)	92 (36.08%)	
2	268 (43.51%)	135 (52.94%)	
3	28 (4.55%)	27 (10.59%)	
4	2 (0.32%)	1 (0.39%)	
S			0.071
0	11 (1.79%)	0	
1	236 (38.31%)	97 (38.04%)	
2	259 (42.05%)	97 (38.04%)	
3	54 (8.77%)	30 (11.76%)	
4	56 (9.09%)	31 (12.16%)	
Significant liver fibrosis	369 (59.90%)	158 (61.96%)	0.572
Advanced liver fibrosis	110 (17.857%)	61 (23.922%)	**0.049**
Significant liver inflammation	298 (48.38%)	163 (63.92%)	**< 0.001**

NAFLD: Non-alcoholic fatty liver disease; G: inflammation grade; S: fibrosis stage

To alleviate the effects of confounders, PSM was performed at a 1:1 ratio, yielding 254 matched pairs. Compared with HBV-infected patients without fatty liver, chronic HBV infection with concurrent NAFLD had higher possibility of having hypertension, T2DM, and had greater body weight, BMI, and greater concentrations of ALT, GGT and ALP (Table 3).

**Table 3:** Clinical characteristics of HBV-infected patients with and without hepatic steatosis after PSM

	HBV infection (N = 254)	HBV infection + NAFLD (N = 254)	P value
Age	39.71 ± 9.33	39.79 ± 9.27	0.332
Gender (M/F)	219/35	219/35	/
Height	167.76 ± 6.61	167.62 ± 7.98	0.796
Weight	64.19 ± 9.27	73.01 ± 10.73	**< 0.001**
BMI	22.79 ± 2.94	25.98 ± 3.56	**< 0.001**
WBC	5.77 ± 1.60	6.01 ± 1.37	0.066
PLT	183.13 ± 53.90	191.55 ± 52.99	0.068
ALB	44.09 ± 3.45	44.53 ± 3.40	0.093
GLB	28.61 ± 3.89	29.03 ± 4.01	0.212
Tbil	16.10 ± 6.50	16.51 ± 6.31	0.456
ALT	41.76 ± 35.98	52.08 ± 37.54	**0.001**
AST	33.46 ± 19.05	35.84 ± 17.61	0.132
GGT	31.25 ± 30.39	42.90 ± 36.33	**< 0.001**
ALP	84.67 ± 21.86	89.56 ± 25.49	**0.015**
HBV DNA (Log_10_ IU/mL)	4.22 ± 2.51	4.25 ± 2.51	0.436
HbeAg (+)	88 (34.65%)	88 (34.65%)	/
Anti-viral therapy	16 (6.30%)	16 (6.30%)	/
Hypertension	12 (4.72%)	32 (12.60%)	**0.002**
T2DM	3 (1.18%)	13 (5.12%)	**0.011**

PSM: Propensity score-matching; NAFLD: Non-alcoholic fatty liver disease; M: Male; F: Female; BMI: Body mass index; WBC: White blood cell count; PLT: Platelet; ALB: Albumin; GLB: Globulin; Tbil: Total bilirubin; ALT: Alanine aminotransferase; AST: Aspartate transferase; GGT: Gamma-glutamyltransferase; ALP: Alkaline phosphatase; HBV: Hepatitis B virus; HbeAg: hepatitis B e antigen; T2DM: Type 2 diabetes mellitus

The histologic characteristics of the patients after PSM were exhibited in Table 4. It revealed that chronic HBV infection with concurrent NAFLD had a greater severity of hepatic steatosis, lobular and portal inflammation, hepatic ballooning and fibrosis. Chronic HBV infection with NAFLD is more prone to have significant inflammation and advanced liver fibrosis, while but the probability of having significant fibrosis between the two groups was similar.

**Table 4:** Histologic characteristics of HBV-infected patients with and without hepatic steatosis after PSM

	HBV infection (N = 254)	HBV infection + NAFLD (N = 254)	P value
Steatosis			**< 0.001**
0	254 (100%)	0	
1	0	218 (85.83%)	
2	0	33 (12.99%)	
3	0	3 (1.18%)	
Lobular inflammation			**< 0.001**
0	1 (0.39%)	0	
1	193 (75.98%)	150 (59.06%)	
2	59 (23.23%)	92 (36.22%)	
3	1 (0.39%)	12 (4.72%)	
4	0	0	
Portal inflammation			**< 0.001**
1	165 (64.96%)	100 (39.37%)	
2	81 (31.89%)	127 (50.00%)	
3	8 (3.15%)	26 (10.24%)	
4	0	1 (0.39%)	
Hepatic ballooning			**0.001**
0	253 (99.61%)	238 (93.70%)	
1	1 (0.39%)	9 (3.54%)	
2	0	7 (2.76%)	
3	0	0	
G			**< 0.001**
1	148 (58.27%)	92 (36.22%)	
2	98 (38.58%)	134 (52.76%)	
3	8 (3.15%)	27 (10.63%)	
4	0	1 (0.39%)	
S			**0.010**
0	9 (3.54%)	0	
1	105 (41.34%)	97 (38.19%)	
2	100 (39.37%)	97 (38.19%)	
3	20 (7.87%)	29 (11.42%)	
4	20 (7.87%)	31 (12.20%)	
Significant liver fibrosis	140 (55.12%)	157 (61.81%)	0.126
Advanced liver fibrosis	40 (15.75%)	60 (23.62%)	**0.034**
Significant liver inflammation	106 (41.73%)	162 (63.78%)	**< 0.001**

PSM: Propensity score-matching; NAFLD: Non-alcoholic fatty liver disease; G: inflammation grade; S: fibrosis stage

### Factors associated with significant liver inflammation, significant fibrosis and advanced liver fibrosis

Univariate analysis showed hepatic steatosis, ALT, AST, GGT, ALB, GLB, PLT and the positivity of HBeAg were predictors of significant liver inflammation. The above characteristics entered into the subsequent multivariate analysis, revealing that the following seven characteristics were associated with higher odds of having significant inflammation in chronic HBV infection: steatosis, decreased ALT and ALB, elevated AST, GGT and GLB, and the positivity of HBeAg (Table 5).

**Table 5:** Factors associated with significant liver inflammation

Variables	Univariate	Multivariate			
	P value	β ± SE	Wald	OR (95% CI)	P value
Age	0.544				
Gender	0.690				
HBV DNA (Log10 IU/mL)	0.863				
HbeAg (+)	**0.031**	**0.436 ± 0.157**	**7.760**	**1.547 (1.138–2.103)**	**0.005**
BMI	0.242				
Hypertension	0.377				
T2DM	0.756				
WBC	0.105				
PLT	**0.053**	-0.003 ± 0.001	3.644	0.997 (0.994-1.000)	0.056
ALB	**0.003**	**-0.080 ± 0.023**	**11.827**	**0.923 (0.881–0.966)**	**0.001**
GLB	**0.013**	**0.057 ± 0.021**	**7.602**	**1.059 (1.017–1.103)**	**0.006**
Tbil	0.082				
ALT	**0.038**	**-0.013 ± 0.005**	**5.500**	**0.987 (0.977–0.998)**	**0.019**
AST	**< 0.001**	**0.054 ± 0.012**	**21.319**	**1.055 (1.031–1.079)**	**< 0.001**
GGT	**0.041**	**0.008 ± 0.004**	**4.606**	**1.008 (1.001–1.015)**	**0.032**
ALP	0.178				
Steatosis	**0.001**	**0.750 ± 0.176**	**18.210**	**2.117 (1.500-2.988)**	**< 0.001**

HBV: Hepatitis B virus; HbeAg: hepatitis B e antigen; BMI: Body mass index; T2DM: Type 2 diabetes mellitus; WBC: White blood cell count; PLT: Platelet; ALB: Albumin; GLB: Globulin; Tbil: Total bilirubin; ALT: Alanine aminotransferase; AST: Aspartate transferase; GGT: Gamma-glutamyltransferase; ALP: Alkaline phosphatase

Both univariate and multivariate analyses demonstrated associated factors for higher probability of significant fibrosis in chronic HBV infection were as follows: decreased levels of HBV DNA, hypertension, decreased ALT, elevated AST, GGT and GLB (Table 6).

**Table 6:** Factors associated with significant liver fibrosis

Variables	Univariate	Multivariate			
	P value	β ± SE	Wald	OR (95% CI)	P value
Age	0.196				
Gender	0.211				
HBV DNA (Log10 IU/mL)	**0.004**	**-0.127 ± 0.029**	**19.317**	**0.881 (0.832–0.932)**	**< 0.001**
HbeAg (+)	0.706				
BMI	0.769				
Hypertension	**0.028**	**0.890 ± 0.341**	**6.802**	**2.434 (1.247–4.751)**	**0.009**
T2DM	0.727				
WBC	0.194				
PLT	0.086				
ALB	0.712				
GLB	**0.037**	**0.040 ± 0.020**	**3.977**	**1.041 (1.001–1.083)**	**0.046**
Tbil	0.952				
ALT	**0.002**	**-0.017 ± 0.005**	**12.163**	**0.983 (0.974–0.993)**	**< 0.001**
AST	**< 0.001**	**0.045 ± 0.010**	**19.461**	**1.046 (1.025–1.067)**	**< 0.001**
GGT	**0.059**	**0.007 ± 0.003**	**4.711**	**1.007 (1.001–1.083)**	**0.030**
ALP	0.952				
Steatosis	0.784				

HBV: Hepatitis B virus; HbeAg: hepatitis B e antigen; BMI: Body mass index; T2DM: Type 2 diabetes mellitus; WBC: White blood cell count; PLT: Platelet; ALB: Albumin; GLB: Globulin; Tbil: Total bilirubin; ALT: Alanine aminotransferase; AST: Aspartate transferase; GGT: Gamma-glutamyltransferase; ALP: Alkaline phosphatase

Table 7 showed factors related to higher risk of advanced liver fibrosis in chronic HBV infection included male gender, decreased levels of HBV DNA, decreased levels of PLT and ALT, and elevated levels of AST, GGT and GLB.

**Table 7:** Factors associated with advanced liver fibrosis

Variables	Univariate	Multivariate			
	P value	β ± SE	Wald	OR (95% CI)	P value
Age	0.067				
Gender (if male)	**0.036**	**0.644 ± 0.232**	**7.720**	**1.904 (1.209–2.998)**	**0.005**
HBV DNA (Log10 IU/mL)	**0.001**	**-0.139 ± 0.038**	**13.350**	**0.871 (0.808–0.938)**	**< 0.001**
HbeAg (+)	0.099				
BMI	0.465				
Hypertension	0.560				
T2DM	0.576				
WBC	0.554				
PLT	**< 0.001**	**-0.010 ± 0.002**	**24.919**	**0.990 (0.987–0.994)**	**< 0.001**
ALB	0.903				
GLB	**< 0.001**	**0.105 ± 0.024**	**19.018**	**1.111 (1.059–1.164)**	**< 0.001**
Tbil	0.338				
ALT	**0.031**	**-0.011 ± 0.005**	**5.384**	**0.989 (0.980–0.998)**	**0.020**
AST	**0.007**	**0.023 ± 0.008**	**7.211**	**1.023 (1.006–1.040)**	**0.007**
GGT	**0.005**	**0.010 ± 0.003**	**9.924**	**1.010 (1.004–1.016)**	**0.002**
ALP	0.533				
Steatosis	0.229				

HBV: Hepatitis B virus; HbeAg: hepatitis B e antigen; BMI: Body mass index; T2DM: Type 2 diabetes mellitus; WBC: White blood cell count; PLT: Platelet; ALB: Albumin; GLB: Globulin; Tbil: Total bilirubin; ALT: Alanine aminotransferase; AST: Aspartate transferase; GGT: Gamma-glutamyltransferase; ALP: Alkaline phosphatase

### Liver histology based on the natural history of chronic HBV infection

The 818 patients who haven’t receive any antiviral therapy were divided into four disease phases of chronic HBV infection according to their clinical data. Among them, 238 patients had concurrent NAFLD, while the other 580 did not. We compared the presence of significant liver inflammation, significant fibrosis and advanced liver fibrosis between the two groups with or without NAFLD according to the natural history of chronic HBV infection. Patients with normal ALT (chronic HBV infection with no evidence of CHB) and elevated ALT values (CHB) were also analyzed separately.

As shown in Table 8, in patients with HBeAg-positive and HBeAg-negative chronic HBV infection (immune tolerant and inactive carrier phase), that is, in those HBV-infected patients with persistent normal ALT value, the presence of significant liver inflammation was higher in NAFLD group than that in the non-NAFLD group (68.750% vs. 43.571%, *P* = 0.011; 59.302% vs. 35.685%, *P* < 0.001; 61.864% vs. 38.583, *P* < 0.001 in chronic HBV infection with no evidence of CHB). Furthermore, in patients with HBeAg-positive chronic HBV infection (immune tolerant phase), the presence of advanced liver fibrosis was higher in NAFLD group than that in the non-NAFLD group (25% vs. 10.714%, *P* = 0.044).

**Table 8:** Liver histology based on the natural history of chronic HBV infection

Phase of chronic HBV infection	NAFLD or not	Total	Significant liver inflammation (%)	*P* value	Significant liver fibrosis (%)	*P* value	Advanced liver fibrosis (%)	*P* value
HBeAg-positive chronic HBV infection	Non-NAFLD	140	61 (43.571)	**0.011**	66 (47.143)	0.434	15 (10.714)	**0.044**
	NAFLD	32	22 (68.750)		18 (56.25)		8 (25)	
HBeAg-positive CHB	Non-NAFLD	111	78 (70.270)	0.455	64 (57.658)	1.000	18 (16.216)	0.508
	NAFLD	51	39 (76.471)		29 (56.863)		11 (21.569)	
HBeAg-negative chronic HBV infection	Non-NAFLD	241	86 (35.685)	**< 0.001**	150 (62.241)	1.000	44 (18.257)	0.749
	NAFLD	86	51 (59.302)		53 (61.628)		17 (19.767)	
HBeAg-negative CHB	Non-NAFLD	88	57 (64.773)	0.325	61 (69.318)	0.397	19 (21.591)	0.848
	NAFLD	69	39 (56.522)		43 (62.319)		16 (23.188)	
Chronic HBV infection with no evidence of CHB	Non-NAFLD	381	147 (38.583)	**< 0.001**	216 (56.693)	0.524	59 (15.486)	0.160
	NAFLD	118	73 (61.864)		71 (60.169)		25 (21.186)	
CHB	Non-NAFLD	199	135 (67.839)	0.625	125 (62.814)	0.636	37 (18.593)	0.471
	NAFLD	120	78 (65)		72 (60)		27 (22.5)	

NAFLD: Non-alcoholic fatty liver disease; HbeAg: hepatitis B e antigen; CHB: chronic hepatitis B

### Subgroup analysis based on metabolic factors

To increase the strength of the correlation between the virus and NAFLD, we make a list of subgroup analyses (Table 9). Firstly, patients with T2DM, hypertension and overweight (defined as BMI ≥ 23 kg/m^2^) were excluded (Subgroup 1). In this subgroup of a total of 409 patients, 41 had concurrent NAFLD, while the other 368 did not. The presence of significant liver inflammation in HBV-infected patients with NAFLD but no metabolic factors is borderline higher than those without NAFLD (60.976% vs. 45.109%, *P* = 0.069), but the prevalence of significant or advanced liver fibrosis between the two groups was comparable.

**Table 9:** Subgroup analysis based on metabolic factors

Subgroups	NAFLD or not	Total	Significant liver inflammation (%)	*P* value	Significant liver fibrosis (%)	*P* value	Advanced liver fibrosis (%)	*P* value
Subgroup 1	Non-NAFLD	368	166 (45.109)	0.069	212 (57.609)	0.623	59 (16.033)	1.000
	NAFLD	41	25 (60.976)		22 (53.659)		6 (14.634)	
Subgroup 2	Non-NAFLD	377	170 (45.093)	**0.035**	218 (57.825)	0.871	62 (16.446)	0.670
	NAFLD	43	27 (62.791)		24 (55.814)		8 (18.605)	

NAFLD: Non-alcoholic fatty liver disease; Subgroup 1: individuals with T2DM, hypertension and overweight were excluded; Subgroup 2: only lean HBV-infected subjects were included

We observed the presence of steatosis in lean individuals (BMI < 23 kg/m2) with HBV infection in this study was approximately 10%, similar with the overall prevalence of steatosis in lean individuals according to literature [15]. There were 420 lean HBV-infected subjects totally, and liver histology was compared in the NAFLD and non-NAFLD group (Subgroup 2). NAFLD group had higher prevalence of significant liver inflammation than the non-NAFLD group (62.791% vs. 45.093%, *P* = 0.035), but the presence of significant or advanced liver fibrosis between the two groups was similar.

### Noninvasive models for significant liver inflammation and significant fibrosis

All included patients with and without significant liver inflammation or significant fibrosis were randomly selected with a ratio of 1:1. 80% of the patients were included in the derivation cohort, while the other 20% were included in the validation cohort.

**Fig. 1:**
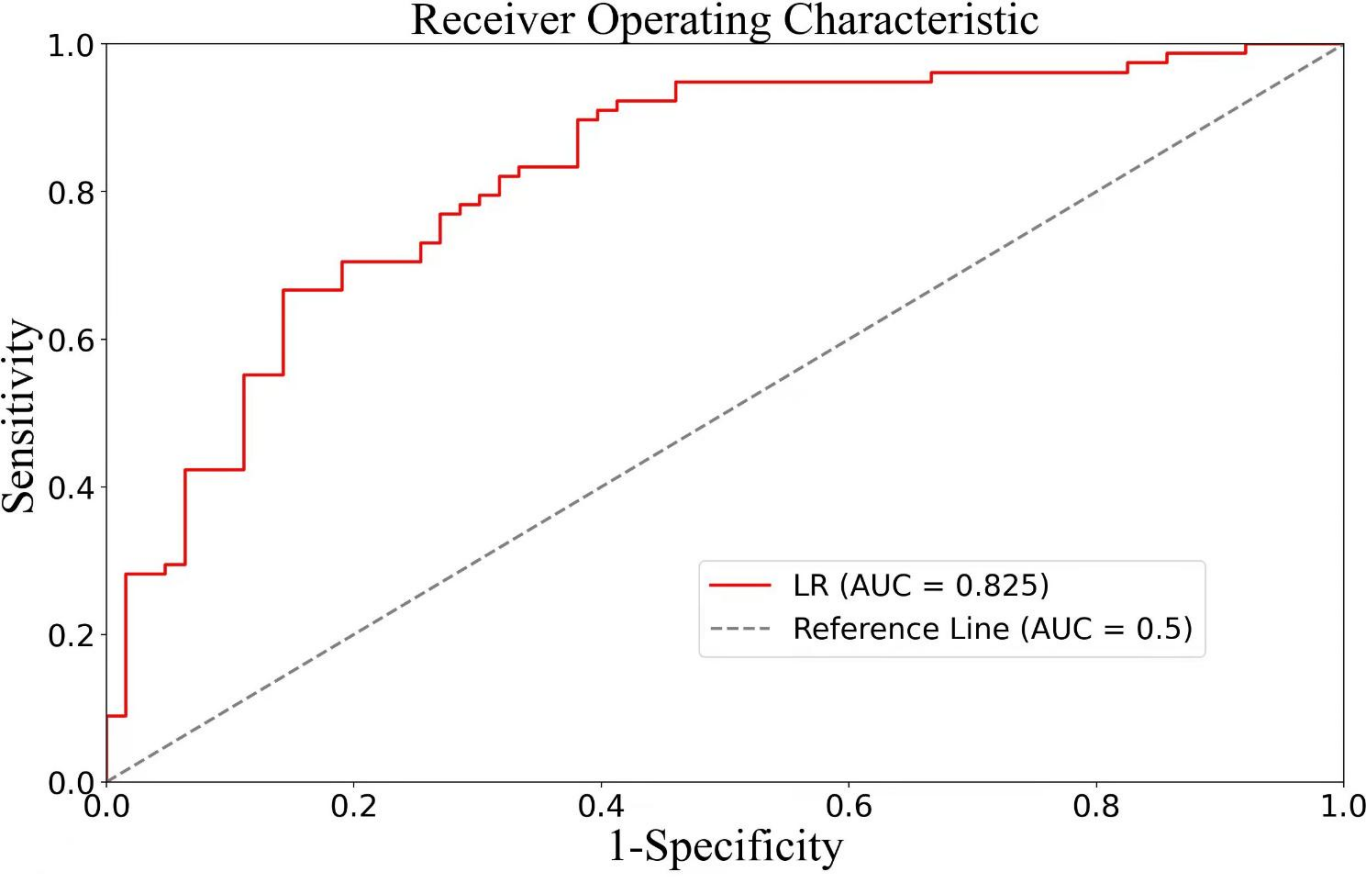
Receiver-operating characteristic (ROC) curves of the predictive model for significant liver inflammation, with the area under the ROC curve of 0.825

The derivation cohort for significant inflammation was composed of 656 patients. Model score = 0.00592 + 0.86997 * AST − 0.40814 * ALT + 0.19083 * GGT − 0.32590 * ALB + 0.29954 * GLB + 0.31760 * (if steatosis) + 0.17445 * (if HbeAg positive) − 0.16393 * PLT. The AUROC of the model was 0.735. In the validation cohort of 164 patients, the AUROC was 0.825 (Fig. 1). At a cut-off value of 0.478, the model could determine significant hepatic inflammation with a sensitivity of 0.705, a specificity of 0.810, and a diagnostic accuracy of 0.758.

**Fig. 2:**
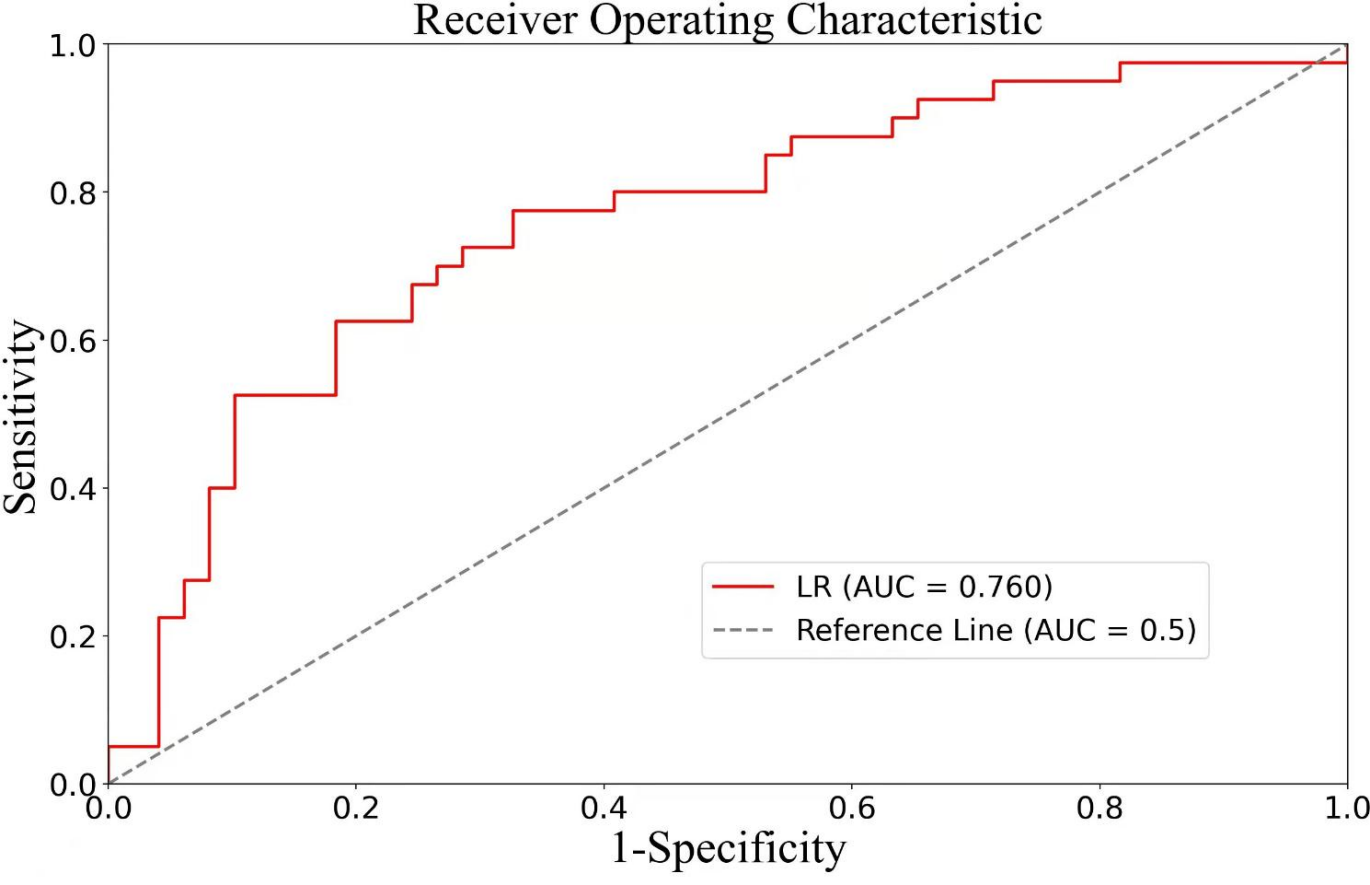
Receiver-operating characteristic (ROC) curves of the predictive model for significant fibrosis, with the area under the ROC curve of 0.760

The derivation cohort for significant fibrosis was consisted of 555 patients. A model was developed with an AUROC of 0.702. Model score = 0.08888 + 0.20617 * (if hypertension) − 0.16412 * ALT + 0.32559 * AST + 0.45615 * GGT + 0.24697 * GLB − 0.41932 * Log10 HBV DNA. In the validation cohort of 110 patients, the AUROC was 0.760 (Fig. 2). At a cut-off value of 0.500, the model could diagnose significant fibrosis in CHB with a sensitivity of 0.725, a specificity of 0.714, and a diagnostic accuracy of 0.719.

## Discussion

The current study highlighted the pathological findings in HBV-infected patients with and without concurrent NAFLD. HBV-infected patients with fatty liver had a higher severity of hepatic steatosis, hepatic inflammation, hepatic ballooning and a higher probability of advanced liver fibrosis than patients with simple chronic HBV infection. Fatty liver was not a risk factor for significant or advanced fibrosis, but it could independently predict significant liver inflammation in chronic HBV infection. Especially, in patients with either HBeAg-positive or HBeAg-negative chronic HBV infection, that is, in HBV-infected patients with persistent normal ALT, the presence of significant liver inflammation was higher in NAFLD than those without NAFLD. The prevalence of advanced liver fibrosis was higher in NAFLD than non-NAFLD group only in HBeAg-positive chronic HBV infection, while NAFLD did not increase fibrosis burden in other stages of HBV infection. Furthermore, we developed noninvasive models for significant liver inflammation and significant fibrosis with good diagnostic performance.

In 2020, a group of experts suggested the nomenclature of NAFLD should be updated to metabolic associated fatty liver disease (MAFLD) [16, 17], because metabolic liver disease usually coexists with other conditions such as viral hepatitis, and should not be described as a condition of “exclusion” [18]. We need to pay attention to patients with dual aetiology fatty liver disease who meet the criteria of NAFLD and who also have other concomitant condition. It is generally recognized that both NAFLD and HBV infection are common types of chronic hepatitis, and both can cause cirrhosis, hepatic failure and hepatocellular carcinoma [19, 20]. The coexistence of NAFLD and chronic HBV infection happens frequently. Therefore, it’s of great importance to explore the relationship between hepatic steatosis and HBV infection.

The interaction between NAFLD and HBV infection is complex and unclear. HBV infection might be related to decreased risk of NAFLD [2, 21], but the mechanisms whereby HBV influences steatosis has not been well understood. On the other hand, how hepatic steatosis influences the clinical outcomes of chronic HBV infection is not entirely clear. Wong et al. has found fatty liver, measured by controlled attenuation parameter (CAP), is associated with advanced fibrosis [6]. Mak’s study demonstrated hepatic steatosis promoted fibrosis progression in virologically quiescent CHB [22]. Another study by Seto et al. revealed that severe steatosis was related to severe fibrosis in both treatment-naive and on-treatment patients with HBV infection [5]. However, the definition of liver fibrosis in the above studies was determined by liver stiffness measurement (LSM) under transient elastography. As we know, moderate to severe hepatic steatosis might result in overestimation of LSM in HBV-infected patients, which should be considered seriously to avoid misdiagnosing fibrosis [23]. Therefore, histology by liver biopsy is always the golden standard. Even though in studies reporting the relationship between concomitant NAFLD and the severity of liver histology in patients based on liver biopsy, the results were not consistent [3, 4, 7].

In this study, we found that HBV-infected patients with concomitant NAFLD had a higher severity of portal inflammation and hepatic ballooning, and were more likely to develop significant liver inflammation and advanced fibrosis (borderline difference). To lighten the effects of confounding factors including age, gender and virological profiles, we performed further evaluation in 254 matched pairs. The results showed that chronic HBV infection with concurrent NAFLD had a greater severity of lobular and portal inflammation, hepatic ballooning and fibrosis, and were more likely to have significant inflammation and advanced fibrosis than simple HBV infection. But the probability of having significant fibrosis between the two groups was similar. Multivariate logistic analyses identified that steatosis was an independent predictor for only significant liver inflammation but not significant or advanced fibrosis in chronic HBV infection. While histologic alterations represent a continuous process, and persistent liver inflammation will definitely lead to the progression of fibrosis [24], it’s better to carry out a longitudinal cohort study to explore whether concomitant NAFLD in HBV-infection could promote liver fibrosis.

As we know, the natural history of HBV infection follows four disease phases with different levels of viral replication and dynamics in liver disease progression. As reported in this study, the influence of concurrent NAFLD on liver histology is different in distinct clinical phases in chronic HBV infection. Briefly, NAFLD could aggravate liver inflammation in HBV-infected patients with persistent normal ALT (immune tolerant and inactive carrier phase), and NAFLD increased the burden of advanced fibrosis only in the immune tolerant phase. This is a pretty novel and interesting finding. We should further explore the inside mechanism relating the interaction between NAFLD and distinct clinical phases in chronic HBV infection.

HBV replication and subsequent liver inflammation and fibrosis account for disease progression in CHB. Significant liver necroinflammation (grade G ≥ 2) and significant fibrosis (stage S ≥ 2) by liver histology greatly increase the risk of cirrhosis, hepatocellular carcinoma and end-stage liver diseases. As a consequence, early and timely antiviral therapy is recommended in these situations. Nevertheless, as the only golden standard for evaluating significant liver inflammation and fibrosis, liver biopsy is limited in clinical practice due to its invasive nature. Current noninvasive predictions of liver fibrosis mainly include LSM and biomarkers like Chitinase 3-like 1 (CHI3L1) [25, 26], Golgi protein 73 [27], et al. The detection of the newly-developed noninvasive markers has not yet been applied widely and their diagnostic accuracies are still limited. LSM could be impacted by a variety of factors like BMI, waist circumference, fatty liver, skin capsular distance and so on [28, 29]. Central obesity and fatty liver will lead to an overestimation of LSM in viral hepatitis including CHB [23] and chronic hepatitis C [30]. Given the gradually increasing prevalence and severity of NAFLD in HBV-infected patients, it’s necessary and has always been a research hotspot to develop noninvasive diagnostic models for significant liver inflammation and fibrosis. Our models were based on routine widely available clinical and laboratory parameters, and have presented with good diagnostic performance.

Despite our best effort, there were still some limitations in this study. Firstly, it was limited to a cross-sectional study, which was unable to evaluate the clinical outcomes of patients with concurrent NAFLD with HBV infection. Long-period follow-up and repeated liver biopsy are of great necessity to understand the influence of progression or regression of fatty liver on the histologic characteristics and clinical outcomes of the disease. Secondly, lack of data on more metabolic factors such as waist circumference and insulin resistance could also affect the interpretation of results. Last but not least, the mechanism behind interactions between NAFLD and HBV infection have not been explored in this study. As literatures report that specific diet and drugs can prevent the progression of chronic HBV infection and NAFLD [31, 32], further mechanism research is warranted in the purpose of reducing the synergistic negative effects of HBV and NAFLD, alleviating liver injury, and preventing decompensation events.

## Conclusions

In conclusion, HBV-infected patients with concomitant NAFLD had a higher severity of liver inflammation, and a higher probability of significant liver inflammation and advanced fibrosis than simple chronic HBV infection. Hepatic steatosis could independently predict significant liver inflammation in nondrinker patients with chronic HBV infection, but it was not a risk factor for significant or advanced fibrosis. NAFLD could aggravate liver inflammation in the immune tolerant and inactive carrier phase of HBV infection, and increase the burden of advanced fibrosis only in the immune tolerant phase. In addition, noninvasive models established in this study could efficiently predict significant liver inflammation and significant fibrosis in patients with chronic HBV infection.

## Data Availability

The datasets used and/or analyzed during the current study are available from the corresponding author on reasonable request.

## List of abbreviations

NAFLD: Non-alcoholic fatty liver disease
CHB: Chronic hepatitis B
HBV: Hepatitis B virus
HBeAg: Hepatitis B e antigen
HBV DNA: Hepatitis B virus DNA
ALT: Alanine aminotransferase
ULN: Upper limit of normal
SD: Standard deviation
PSM: Propensity score-matching
AUROC: Area under receiver operating characteristic curve
T2DM: Type 2 diabetes mellitus
WBC: White blood cells
BMI: Body mass index
PLT: Platelet
ALB: Albumin
GLB: Globulin
Tbil: Total bilirubin
AST: Aspartate transferase
GGT: Gamma-glutamyltransferase
ALP: Alkaline phosphatase
MAFLD: Metabolic associated fatty liver disease
CAP: Controlled attenuation parameter
LSM: Liver stiffness measurement
CHI3L1: Chitinase 3-like 1
ROC: Receiver-operating characteristic
